# STATCOM Estimation Using Back-Propagation, PSO, Shuffled Frog Leap Algorithm, and Genetic Algorithm Based Neural Networks

**DOI:** 10.1155/2018/6381610

**Published:** 2018-04-26

**Authors:** Hamed Atyia Soodi, Ahmet Mete Vural

**Affiliations:** Electrical and Electronics Engineering Department, University of Gaziantep, Şahinbey, 27310 Gaziantep, Turkey

## Abstract

Different optimization techniques are used for the training and fine-tuning of feed forward neural networks, for the estimation of STATCOM voltages and reactive powers. In the first part, the paper presents the voltage regulation in IEEE buses using the Static Compensator (STATIC) and discusses efficient ways to solve the power systems featuring STATCOM by load flow equations. The load flow equations are solved using iterative algorithms such as Newton-Raphson method. In the second part, the paper focuses on the use of estimation techniques based on Artificial Neural Networks as an alternative to the iterative methods. Different training algorithms have been used for training the weights of Artificial Neural Networks; these methods include Back-Propagation, Particle Swarm Optimization, Shuffled Frog Leap Algorithm, and Genetic Algorithm. A performance analysis of each of these methods is done on the IEEE bus data to examine the efficiency of each algorithm. The results show that SFLA outperforms other techniques in training of ANN, seconded by PSO.

## 1. Introduction

The power systems are the backbone of any country's economic and social sectors, without which a country cannot excel in the industrial and social development. But the power systems face the ever-growing load demand as more industrial and housing units are established, which makes the job of power system managing challenging. Recently, the increase of nonlinear loads has badly affected the power quality, due to inherent voltage fluctuations in these types of loads, and has also raised question on the long-term stability of the power systems and their associated instruments [1, 2]. Hence, more research studies have been dedicated to improving the power quality and efficiency through variety of different techniques. The total power in the system contains both real and reactive power, which implies that if the reactive power of the system is improved, the overall system can benefit from this improvement.

A family of different devices which can control the reactive power at designated buses is given the name Flexible AC Transmission Systems (FACTS). These devices have the capability to dynamically adjust different system parameters to enhance the performance and quality [2]. The FACTS are actually controllers which can improve the system stability in terms of voltages, reactive power, and phase angles in the steady-state operation. One of the important FACTS devices which we have focused on in this research is called the Static Synchronous Compensator (STATCOM). A STATCOM is used to control the bus voltage or reactive power injection/absorption at the bus and is connected in shunt with the designated bus.

The STATCOM when used as a voltage regulator draws controllable reactive currents from the buses. Since it is an expensive device, the selection of the optimal bus for the installation is of prime importance. When installed at an ideal location, the STATCOM can improve the efficiency of the power systems significantly [3–6]. However, the STATCOM must be installed on a load bus only, since the generator buses do not need voltage regulation [7–9]. Several authors have reported the use of STATCOM for the voltage and reactive power regulation with different configurations. For example, in larger power systems involving hundreds of buses, multipulse inverters based controllers are used because they provide lower harmonic distortion [5, 10, 11].

The planning, implementation, control, and maintenance of power systems initiate with the power flow calculations which constitute the crux of any power system. Over the past few decades, many different solutions have been proposed for the load flow problems. The most important of these techniques have been reviewed in [12]. At any instant, the power systems experience varying operating conditions and the power flow calculations ensure that the operation of the system is within the bounds of the stability criterion. The power flow equations are complex nonlinear algebraic equations which are usually written in computer programs, which are run over the course of the operation for dynamic analysis. Usually, these equations are solved in multiple iterations and hence require substantial processing and memory requirements [13, 14]. One of the primary methods used for the solutions of nonlinear equations is Newton-Raphson method [15], which is widely known for its quadratic convergence features. However, the conventional load flow studies do not account for the presence of STATCOM device(s) in the system and hence the method must be redesigned for the STATCOM buses. In this paper, we have briefly explained the method to modify the existing load flow equations to incorporate the STATCOM parameters such as reactive powers and voltage sources using Newton-Raphson method as done in [16]. Many research studies have been dedicated to the development of modified models for the STATCOM such as [16–19].

Despite all the benefits of Newton-Raphson method, this method is a complex one and requires large memory and processing capabilities. In real time power systems, power systems analysis including economic load dispatch must be done as frequently as every 5 to 15 minutes [20], which becomes very difficult with classical mathematical approaches. The situation is further aggravated as the huge power systems undergo parameters shifting very rapidly. In order to tune the control parameters of STATCOM, the NR method needs to be run countless times in a system as the system passes through different operational states. This makes the whole calculations hectic and time-consuming. We propose an alternative approach to this method which is based on machine learning algorithms. More specifically, we propose the use of Artificial Neural Network (ANN) for estimating the STATCOM parameters such as voltages, phase angles, and reactive powers. ANN is a very powerful tool which can be used for the data fitting operations as well as classification problems. ANN has been successfully used in different fields [21] which involve use of datasets, such as medical and biomedical applications [22–24], business, finance, stock markets, and foreign exchange [25–28], and power applications [29, 30]. The ANN can be trained to capture the nuances in the input data and to produce estimated outputs accordingly, which in this case would be the estimated voltages and reactive powers. The ANN can be used efficiently in real time power systems to do the load flow analysis much faster than the NR method, thus saving cost of computation power and making shrewd decisions at the right moment.

Three separate ANNs have been developed which take the real and reactive powers of the STATCOM bus and predict three different outputs. First ANN is used to estimate the voltage magnitude and the second uses ANN to find phase angle of the STATCOM bus, while the third and last ANN is used to estimate the reactive power of the STATCOM bus. In order to generate a data set of real and reactive powers of the STATCOM bus, the real and reactive powers of all the load buses in the system were perturbed by gradually increasing their values and the corresponding voltages, angles, and reactive powers at the output were recorded. This data is then fed to both of the ANNs for their respective tasks.

Usually, the Back-Propagation (BP) method is the primary method for training the neural networks; however, this method is prone to get stuck in the local minima and also experiences slower convergence rate towards the optimal solution [33]. Alternative approach to the training of neural network for the optimal weight setting is to use metaheuristic techniques to avoid local minima and slow convergence problems. We have used multiple metaheuristic techniques in this study to tune the weights of the ANN with promising results. A survey of different randomized techniques for the training of neural networks is presented in [34]. First one is the Particle Swarm Optimization technique which is based on stochastic optimization. This technique is based on the mimicking of social behavior of swarm of birds flying over an area in search for food. The birds represent the solutions and the total area over which the birds are flying is the search space, while the food represents the optimal solution in the whole search space [35]. PSO performs better than Back-Propagation for training the neural network in terms of rate of convergence [36–38]. PSO can be applied to improve various aspects of the neural network such as weights assigned to different layers and the number of layers. Several works in literature have used PSO for the purpose of training neural networks including [38], which has used neural network for nonlinear channel equalization. In [39], PSO trained neural network is used to predict structure failure in multistoried RC buildings. Similarly [40] presents a review of different PSO trained ANNs which are used in wind energy systems. In [26], PSO based neural networks are used for the forecasting of foreign exchange rates. Another effort is the use of PSO trained neural network in ground water management, which is used to minimize the operational cost of pumps and pipelines connected to the wells [41]. In geology, PSO based ANN is used to estimate the compressive strength of rock samples [42].

Furthermore, we have also applied Shuffled Frog Leap Algorithm (SFLA) [43] for parameter tuning of the ANN. SFLA is another memetic algorithm inspired by the cooperative search metaphor of frogs. The population (solutions) called frogs is divided into different memeplexes each carrying its own meme. The frogs search for local optima in each memeplex using an evolution method which is comparable to the PSO. In the next stage, the frogs are reshuffled to likely a different memeplex based on their global ranking which is comparable to the shuffled complex evolution algorithm. This ensures that global optima is achieved by the frogs. The SFLA has been proved to be an effective tool in the optimization problems. There are several examples of using SFLA for the training of different types of neural networks such as [44] which uses SFLA to train neural networks which are used in channel equalization and estimation problem. Similarly, [45] has used SFLA to propose three novel techniques for scheduling problem. The authors solve multiprocessor problem in grid environment by using SFLA directly, followed by training the ANN and Radial Basis Function Neural Network (RBFNN) using SFLA. SFLA is also used in acoustics such as [46], which has trained wavelet neural network to locate the source of acoustic emission in rotating machinery to diagnose the friction fault source. In [47], the authors have proposed a combination of Improved SFLA (ISFLA) and Back-Propagation to train the neural network to diagnose early faults in rolling bearings.

At last, but not the least, Genetic Algorithm [48] is also applied for the parameter tuning of the ANN. Genetic Algorithm is another efficient method of optimization which has been vastly used for different problems pertaining to the optimization of different parameters. Genetic Algorithm is based on the Darwinian concept of survival and involves natural selection and natural genetics. The algorithm consists of binary strings which are evolved during the run, on the basis of their probabilities and minimal cost. The algorithm consists of certain operations such as mutation, crossover, and reproduction. Genetic Algorithm is used in literature to provide training to the neural network parameters. This includes [49], which has used GA based ANN to model slump of Ready Mix Concrete (RMC) based on its five ingredients. A combination of GA and ANN is used in [50] to solve the inverse kinematics problem of a six-joint Stanford robotic manipulator. The authors have used three different networks training networks using different training sets. Time series forecasting is an efficient way to analyze the impact of future decisions, both in organizational and individual capacities. The time series has been forecasted using GA based ANN in [51], where automatic design of Artificial Neural Networks (ADANN) is used. In [52], GA and ANN have been used to model and optimize the removal of methylene blue using activated carbon.

In terms of similar work, that is, the use of Newton-Raphson and ANN for estimation of different parameters of power systems, there are several instances. For example, [53] has used PSO tuned ANN to estimate operating conditions of the STATCOM. Specifically, the authors have developed two separate neural networks to estimate the STATCOM voltage and reactive power. Both the neural networks are trained using PSO. The authors perturbed the real and reactive powers to produce larger dataset, used Newton-Raphson method to calculate the voltages and reactive powers, and used ANN to estimate voltages and reactive powers. Quite similarly, the authors of [54] have presented an optimal power flow study using two methods, Newton-Raphson based iterative method and Back-Propagation ANN. The outputs to be estimated include voltages amplitudes, phases, and other parameters.

Further works include [55], which has used ANN for the calculation of causation of anomalies in input data on the outputs in power systems. Specifically, ANN is used to calculate the state of power system based on the input data, which is taken as the real and reactive powers, while the outputs are upper and lower limits of voltage magnitudes and phase angles. In [56], the proper size of STATCOM is calculated in both normal and contingency cases, using Newton-Raphson method.

## 2. Modeling of Power Systems

**Figure 1:**
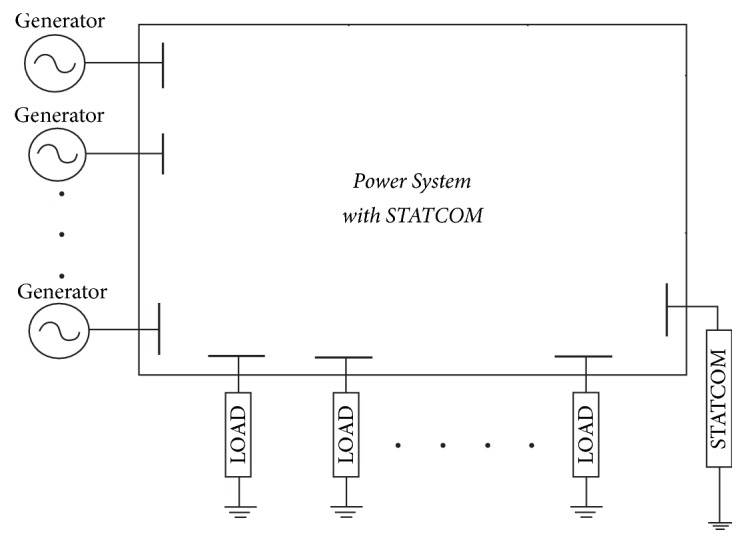
A power system representation with STATCOM.

Each power system consists of several buses interconnected with each other through transmission lines. The buses can either be load or generator buses. The interconnection of different buses can be represented by the admittance matrix or the *Y*-matrix. The *Y*-matrix is a better representation of the transmission lines because most of the entries in this matrix are zero, as compared to the reactance matrix. However, the *Y*-matrix does not incorporate the admittances associated with the load connected to the buses and STATCOM controller. This representation is shown in Figure 1. The steady-state model of the system is represented by the static load flow equations for the real and reactive powers of the buses along with the equality constraints of the transmission network. The static load flow equations of a specific bus are written in terms of voltage magnitudes and phase angles of all the buses connected to this bus. That is, the load flow equations for real (*P*_*i*_) and reactive power (*Q*_*i*_) of bus “*i*” are written as

(1) Pi=∑j=1NViVjYijcos⁡δi−δj−θijQi=∑j=1NViVjYijsin⁡δi−δj−θij.

In these equations, |*V*_*i*_| is the magnitude of the voltage at bus “*i*” and |*V*_*j*_| is the voltage magnitude of “*j*th” bus connected to the bus “*i*,” while “*δ*” represents the corresponding phase angle of the voltage and *N* is the total number of buses in the system. |*Y*_*ij*_| and *θ*_*ij*_ are magnitude and phase angle of the admittance between buses “*i*” and “*j*”. *Y*_*ij*_ can be calculated from the admittance matrix *Y*_Bus_ of the bus system, which is given as

(2) $$\begin{array}{c} Y_{\text{Bus}}=\left[\begin{array}{ccccc} Y11 & Y12 & Y13 & \cdots & Y1N\\ Y21 & Y22 & Y23 & \cdots & Y2N\\ Y31 & Y32 & Y33 & \cdots & Y3N\\ \vdots & \vdots & \vdots & \ddots & \vdots \\ YN1 & YN2 & YN3 & \cdots & YNN \end{array}\right]. \end{array}$$

Here *Y*_*XY*_ = −*y*_*XY*_ is the negative of line admittance from bus *x* to bus *y*, containing the real and imaginary part. *y*_*XY*_ is calculated as

(3) $$\begin{array}{c} y_{XY}=\frac{1}{R}+j\frac{1}{X}=G+jB, \end{array}$$

where “*G*” is the conductance of the line and “*B*” is the susceptance (*R* and *X* being the resistance and reactance). The self-admittance terms *Y*_*XX*_ can be calculated as

(4) YXX=yX0+yX1+yX2+⋯+yXX−1+yXX+1+⋯+yXN.

For each of the load busses, there are two equations for the corresponding real and reactive powers, while there are four unknown variables of voltages and phase angles. Hence, these equations need to be solved using nonlinear iterative methods. The most common method is the Newton-Raphson method, which requires the Jacobian of the equations. The Jacobian of the above equations can be represented as

(5) $$\begin{array}{c} \left[\begin{array}{cc} J^{1} & J^{2}\\ J^{3} & J^{4} \end{array}\right]\left[\begin{array}{c} \Delta \delta \\ \Delta \left|V\right| \end{array}\right]=\left[\begin{array}{c} \Delta P\\ \Delta Q \end{array}\right]. \end{array}$$

In the above equation, sub-Jacobian entries are defined as *J*^1^ = ∂*P*/∂*δ*, *J*^2^ = ∂*P*/∂|*V*|, *J*^3^ = ∂*Q*/∂*δ*, and *J*^4^ = ∂*Q*/∂|*V*|. With the addition of STATCOM, the equations of the bus connected to the STATCOM are slightly modified, which are presented and justified in the next section.

## 3. Power Systems with STATCOM

**Figure 2:**
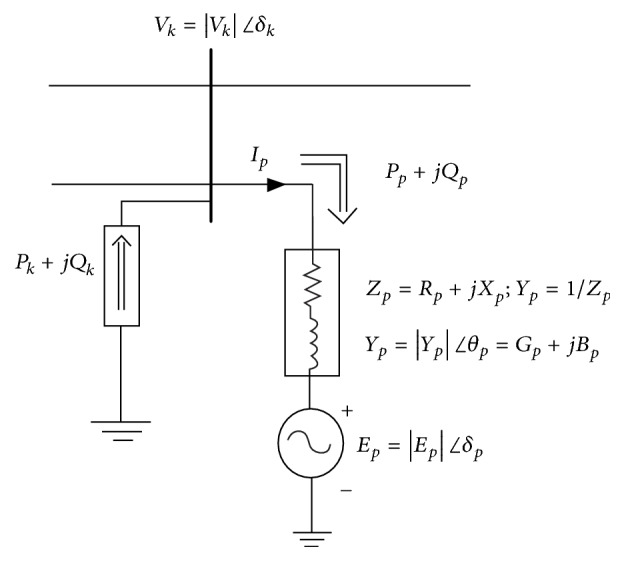
Equivalent diagram of STATCOM [31].

As mentioned in the introduction, the purpose of the STATCOM is to regulate the voltage and reactive powers of the power systems. Hence, the STATCOM has two modes:

- Voltage regulation: in this mode, the STATCOM is used to regulate the reactive power by injecting or absorbing the reactive power to and from the bus, respectively, by incorporating voltage source converter circuit and hence it stabilized the voltages to a predefined value. The STATCOM will absorb the reactive power from the bus when the voltage is higher than the limit and will inject reactive power to the bus when the voltage is below the defined limit.
- VAR control: in this mode, STATCOM keeps the reactive power of the bus at a constant value. The equivalent diagram of the STATCOM in steady-state operation is shown in Figure 2.

The figure shows the model of STATCOM, which is connected to the bus “*k*.” In this setting, the STATCOM is represented by a controllable voltage source with electric potential *E*_*p*_ and phase angle *δ*_*p*_, which is connected in series with an impedance *Z*_*p*_. *Y*_*p*_ is the admittance of the link. Since the admittance is complex, the real part of the impedance models the real power losses (*P*_*p*_) of the devices installed on the system such as converters. The leakage inductances of the coupling transformer makes up for the imaginary part of the reactance power loss (*Q*_*p*_). This STATCOM model is connected in parallel to the bus “*k*,” whose net real and reactive powers are represented as *P*_*k*_ and *Q*_*k*_, respectively. If the STATCOM is being operated in the Voltage Control Mode, it will absorb or inject the reactive power to keep the voltage magnitude of the bus “*k*” (|*V*_*k*_|) constant (usually at 1.0 p.u). With the introduction of STATCOM, the power flow equations are changed only for the bus “*k*,” to which the STATCOM is connected. These equations are represented as

(6) Pk=Pp+∑j=1NVkVjYkjcos⁡δk−δi−θkjQk=Qp+∑j=1NVkVjYkjsin⁡δk−δi−θkj.

Thus, for the bus “*k*,” the original equation is modified by the addition of the real and reactive powers (*P*_*p*_ and *Q*_*p*_) of the STATCOM device. These powers can further be represented as

(7) Pp=GpVk2−VkEpYpcos⁡δk−δp−θpQp=−BpVk2−VkEpYpsin⁡δk−δp−θp.

It is evident from the above equations that STATCOM at bus “*k*” has introduced two new variables, electric potential *E*_*p*_ and phase angle *δ*_*p*_, in the equation set. However, |*V*_*k*_| is now a known variable with a predefined value. Thus, the solution requires one more equation to solve the problem using Newton-Raphson method. By principle, the real power consumed by the STATCOM must be zero in the steady-state operation. The equation for the voltage source power must be equal to zero then; this equation is given as

(8) PEp=RealEpIp∗PEp=−GpEp2−VkEpYpcos⁡δp−δk−θp.

With this modification in the equation of the bus “*k*,” the Jacobian matrix for the updated system is given as

(9) $$\begin{array}{c} \left[\begin{array}{ccc} J^{1} & J^{2} & J^{3}\\ J^{4} & J^{5} & J^{6}\\ J^{7} & J^{8} & J^{9} \end{array}\right]\left[\begin{array}{c} \Delta \delta \\ \Delta \left|V\right|\\ {\Delta \delta }_{p} \end{array}\right]=\left[\begin{array}{c} \begin{array}{c} \Delta P\\ \Delta Q \end{array}\\ {\Delta P}_{p} \end{array}\right]. \end{array}$$

## 4. Overview of Artificial Neural Networks

ANN is an excellent machine learning algorithm for the regression and classification problems, which can estimate the data by emulating the functionality of human brain—it works in different layers each with certain number of neurons and weights. The ANN can be used to estimate nonlinear systems even when the input data is sophisticated and contains redundant and corrupt information. Due to its nonalgorithmic nature, ANN does not try to approximate the solution like the conventional techniques which are used for solving the load flow equations.

A neural network is composed of nodes called* neurons,* at each of which the inputs from previous layers are accumulated after being multiplied with some weights. The neurons are the fundamental processing units which are interconnected with each other in a certain pattern. The human brain comprises trillions of interconnections between the neurons. It is estimated that there are 10 billion neurons present which are interconnected through 10^14^ links [32]. An isolated worthless neuron becomes powerful when interconnected with other neurons in the network. In a similar fashion, Artificial Neural Networks are comprised of interlinked neurons, whose arrangement depends on the type of application. Each neural network has the following basic layers.

*Input Layer*. This layer comprises passive nodes whose sole job is to transmit the input to the next layer and therefore the number of nodes in this layer is equal to the number of inputs to the network. Each node carries specific weight factor, which is multiplied with each input value. The number of neurons in the input layer is represented as matrix with number of rows determined arbitrarily according to the dataset and number of columns being equal to the number of input features.

*Hidden Layer*. This is the most important layer of the network which consists of arbitrary number of sublayers, each containing different number of neurons. This layer processes the data from the input layer by multiplying it with the weight factors.

**Figure 3:**
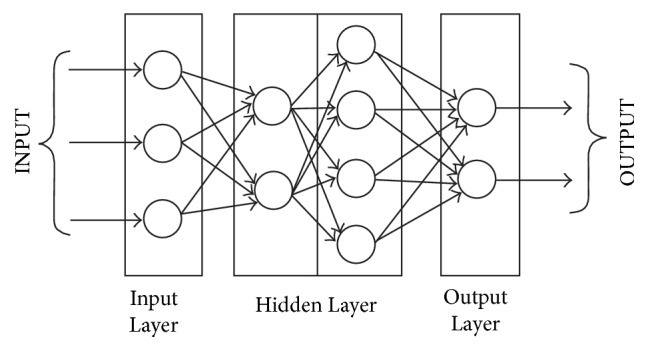
Artificial Neural Network [32].

*Output Layer*. This layer interfaces the internal layers with the outputs and therefore the number of nodes in this layer is equal to the number of outputs. Nodes in this layer are active since they process the data received from the internal layers before transmitting it to the output. A schematic of the connections in ANN is shown in Figure 3.

**Figure 4:**
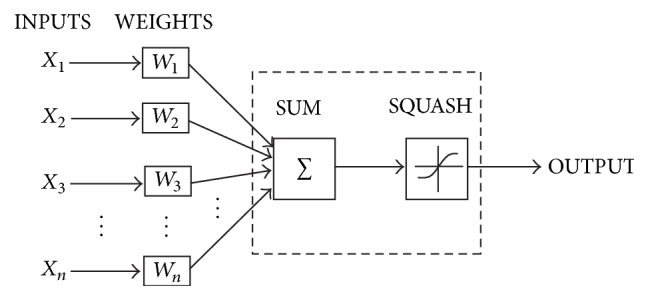
A single neuron structure [32].

This interconnection improves the performance of the system over the traditional numerical analysis schemes. Obviously, the inputs are received in the hidden layer after being multiplied with weights in the input layer. The structure of an artificial neuron is shown in Figure 4. The weighted inputs are summed and passed through the transfer function to produce the output. A transfer function's job is to restrict the widely ranging inputs and map them to a finite output and that's why it is also called “squash.” At each node “*j*,” the weighted-input sum is represented by the equation

(10) $$\begin{array}{c} S_{j}=\overset{n}{\underset{i=1}{\sum }}X_{i}W_{i}. \end{array}$$

The output of the neuron “*j*” is written as “*O*_*j*_”:

(11) $$\begin{array}{c} O_{j}=T_{j}\left(\;S_{j}\;\right). \end{array}$$

One important issue in the design of ANN is the number of hidden layers and neurons in each layer. While the input layer and output layer topology depend largely on the input and output, respectively, hidden layer's topology can be adjusted. Lower number of hidden neurons can result in poor estimation of the function, while higher than required number of neurons might result in overfitting of the network on the training dataset and might also incorporate the effects of noise, apart from increasing the computation time. Therefore, the optimal number of neurons must be decided on an empirical basis. Another way to decide optimum hidden neurons is to introduce self-adaptation in the network, while the number of hidden layers is usually found appropriate and restricted to one in most of the literature [24, 55].

A neural network must be trained for the specific dataset before starting to make its own decisions. The training of the neural network implies fine-tuning of the weights in each layer, such that it is able to produce the expected output with minimum error. Therefore, ANN works in two parts: (i) training and (ii) testing. In training phase, part of the dataset is fed to the ANN along with the determined output. The output obtained from the neural network is compared with the original output (also called target) and the error is fed back to the network to adjust the weights accordingly. When the weights produce optimum result, with minimum error, the ANN is ready to be tested.

The neural networks are usually trained using Back-Propagation method which is a variant of the Least Mean Squares Method due to its activation function which is analytic continuous function. The Back-Propagation method is based on steepest-descent algorithm, which seeks to find the minimum error by adjusting weights in the direction of lowest error. The error is taken and back-calculated to converge to the optimum solution. Thus, in essence, the error propagates in the backward direction and that is why it is called Back-Propagation method. The error at the neuron “*j*” is calculated as

(12) $$\begin{array}{c} E_{j}=\frac{1}{2}{\left(\;R_{j}-O_{j}\;\right)}^{2}. \end{array}$$

The total error “*E*” in all neurons becomes

(13) $$\begin{array}{c} E=\underset{j}{\sum }E_{j}=\frac{1}{2}\underset{j}{\sum }{\left(\;R_{j}-O_{j}\;\right)}^{2}. \end{array}$$

In order to minimize the total error, the weights are adjusted by adding a weight change (ΔW) to the original weights after each iteration. In this respect, a parameter “*α*,” which is the learning rate, is used along with the gradient descent algorithm to define the weight changes as

(14) $$\begin{array}{c} {\Delta W}_{kj}=-\alpha \frac{\partial E}{{\partial W}_{kj}}\;0<\alpha \leq 1. \end{array}$$

This means that if the gradient is positive, the weight change is negative and vice versa, to ensure that the solution converges towards the least error. The solution in the next iteration becomes

(15) $$\begin{array}{c} W_{kj}^{\prime }=\Delta W_{kj}+W_{kj}. \end{array}$$

However, Back-Propagation sometimes gets stuck in the local minimum. One solution is to use this technique with other methods such as the one proposed in [57]. Another solution is to use metaheuristic methods to fine-tune the neural networks.

Another important question is the validity of the use of ANN over Newton-Raphson method. The major advantage of ANN over NR is that NR is an iterative method which takes valuable amount of time to solve the unknown variables in the system. The necessary element of NR method is Jacobian matrix, whose dimensions grow as the system variables increase. As mentioned previously, the huge power systems are dynamic in nature and therefore load flow analysis is critical after every few minutes to few seconds. The use of NR might be burdensome since it consumes time and computation power. In contrast, the ANN has the advantage that it is nonparametric and useful in modeling nonlinearities. Moreover, they can be efficiently implemented on multiprocessor architectures due to their nature and thus they can drastically reduce the processing time [58]. Having said this, it should be mentioned that processing time and power in ANN is required only during the training phase of the network. Once the ANN is trained, it can estimate the required output without much effort and this is the fundamental advantage of using ANN in place of NR in load flow analysis.

## 5. Overview of Optimization Techniques

In this section, different optimization techniques are explained. As described earlier, these optimization techniques are used to train the weights of the neural network, such that the estimation error is reduced considerably.

### 5.1. Particle Swarm Optimization (PSO)

Particle Swarm Optimization is an excellent optimization technique which can be used to tune the weights of the ANN. PSO finds the best solution by allowing different particles to converge to their own best solution (called *P*_best_) and the best solution of the entire population (called *G*_best_). Each particle* “i”* can be can be represented in D-dimensional space as *X*_*i*_ = (*x*_*i*1_, *x*_*i*2_,…, *x*_*iD*_). As mentioned previously, the PSO adjusts the direction of each particle in the search space by regulating the velocity of each particle, which is calculated on the basis of its own best experience (known as cognitive learning) as well as the experience of the best particle in the entire population (called social learning). If the velocity at iteration “*k*” is represented by “*v*(*k*)” and particle's current position by “*x*(*k*),” then the velocity in the next iteration is calculated using the equation

(16) vik+1=wi.vik+c1·rand·Pbest−xik+c2·rand·Gbest−xik.

where *P*_best_ = (*p*_*i*1_, *p*_*i*2_,…, *p*_*iD*_) represents the local best solution of particle “*i*,” *G*_best_ = (*g*_1_, *g*_2_,…, *g*_*D*_) is global best solution of entire swarm, “*w*” is the inertia constant, “*c*_1_” is the cognitive constant and *c*_2_ is the social constant, and rand() function generates a random number between 0 and 1. All of these constants have specific roles in determining the rate of convergence. In one study [58], the optimum values of these parameters are calculated as *c*_1_ = *c*_2_ = 1.494 and *w* = 0.729.

After the calculation of velocity for each particle in the next iteration, the position of the particle “*i*” is updated according to the equation:

(17) $$\begin{array}{c} X_{i}\left(\;k+1\;\right)={v_{i}\left(k+1\right)+X}_{i}\left(\;k\;\right). \end{array}$$

One important aspect in PSO is the population or swarm size. The original authors of PSO have proposed in [59] that a swarm size of 20–100 particles usually produces similar results; however, the size of swarm should also be adjusted based on the dataset dimensions, constraints, and cost function [60].

*Shuffled Frog Leap Algorithm (SFLA)*. Shuffled frog leap algorithm (SFLA) [43] is another population based metaheuristic which works in a way which closely resembles the PSO. This method converges to the optimal solution by evolution of memes which are carried by the particles (called frogs in this regime), which exchange information with each other. Each frog has an associated cost and, during each iteration, the frogs try to improve their cost. The frogs are the carrier of memes which consist of memotypes. This algorithm combines both deterministic and random approaches. In deterministic part, the algorithm uses response surface information to search for the optimal solution. On the other hand, the random part allows the algorithm to instill robustness and flexibility. Initially, the algorithm generates random solutions (frogs) just like PSO. The cost of all the frogs is calculated and frogs are ranked according to ascending order of their cost. Then, starting from the top position, the frogs are partitioned into communities called memeplexes. Within each of these memeplexes, the frogs share their memes or ideas with other frogs and therefore each memeplex evolves on its own in terms of the cost. In each memeplex, the frogs with the best and worst costs (*P*_*B*_ and *P*_*w*_) are noted. The next step is to improve the position of the worst frog (*P*_*w*_). The new position of the worst frog is updated as

(18) $$\begin{array}{c} U=P_{w}+S. \end{array}$$

“*S*” defines the step size (similar to the velocity in PSO) and it is calculated as

(19) S=min⁡rand∗PB−PW,Smax⁡for  positive  stepS=max⁡rand∗PB−PW,−Smax⁡for  negative  step.

*S*_max⁡_ is the maximum step size allowed and rand generates a random number between 0 and 1. At each iteration, the worst frog is moved close to the best frog and the cost of the new position U is calculated. If the cost is better than the previous cost, the new position is assigned to the worst frog; else another step size is calculated as

(20) S=min⁡rand∗PX−PW,Smax⁡for  positive  stepS=max⁡rand∗PX−PW,−Smax⁡for  negative  step.

Here *P*_*X*_ is the position of the global best frog in all memeplexes. Once again the new position's cost is compared with the worst frog's cost. If it is better, the new position replaces the old worst frog; else this frog is terminated and another random frog is generated. After predefined number of iterations, the frogs in the memeplexes are reshuffled. In this way, memeplexes become richer with new ideas and quality of the meme gets improved.

The important parameters in SLFA are the total number of memeplexes and number of frogs in each memeplex. As the size of each memeplex increases, the probability of converging to the global optima also increases; however, this also increases the computational demand. As proposed in [43], the number of memeplexes can be set in the range of 10–100, while the number of frogs in each memeplex can vary from 5 to 300. However, it is imperative that optimum values for these parameters can be obtained through testing the algorithm with different combinations.

### 5.2. Genetic Algorithm (GA)

Genetic Algorithm proposed by Goldberg and Holland [48] is another very efficient optimization algorithm. This algorithm is based on the natural selection and genetics which are derived from the Darwinian concept of survival. The solutions consist of binary strings with exchange information with each other through different operations. Usually, the solutions are represented in terms of binary strings. The initial solutions are randomly generated and their cost is calculated. The Genetic Algorithm performs three basic operations on these solutions.

*Reproduction* is the process of copying the strings according to their fitness, which implies that higher fitness incurs more chances of survival.

*Crossover *is the process of probabilistically choosing two parents of a new solution (called child) based on their fitness value. Furthermore, the crossover site is also chosen at which the two parents are split.

*Mutation* randomly changes the bit values of the newly born child, because there are chances that a bit might remain unchanged by previous operations. This function can also be used to modify the newly born children which are not feasible.

The above processes are repeated in each iteration, allowing the solutions to evolve and mature. At each iteration, the previous solutions are replaced by the new solution. When the termination condition is met, the solution with the best cost is taken as the final solution.

Regarding the initial population of Genetic Algorithm solutions, different theories have been suggested, including dependency on difficulty or building blocks in the problem [61, 62] and self-adaptation [63]. However, the simplest approach to the initialization of population is the empirical method, that is, testing the GA with different number of initial solutions and using only correct number of solutions after testing. This approach is corroborated by many studies such as [64, 65], because, most of the times, the problem is difficult to characterize in terms of difficulty [66].

### 5.3. Cost Function

Since this work is dedicated to the study of different estimation techniques, the difference between the actual and estimated output is the actual error or cost of the system. The cost function can be represented in mathematical form as

(21) $$\begin{array}{c} f\left(\;x_{i}\left(\;k\;\right)\;\right)=E_{i}\left(\;k\;\right). \end{array}$$

The cost function “*f*(*x*_*i*_(*k*))” is the cost of the “*i*th” particle in the “*k*th” iteration of the optimization algorithm. The error term *E*_*i*_(*k*) is taken as the mean-squared error between the original and estimated output, which is the second moment about the origin and is given as follows [67]:

(22) $$\begin{array}{c} E_{i}\left(\;k\;\right)=\frac{1}{N}\overset{N}{\underset{n=1}{\sum }}\left(\;y_{d}\left(\;n\;\right)-\hat{y}{\left(\;n\;\right)}^{2}\;\right), \end{array}$$

where *N* is the total samples of datasets which are applied to the network, *y*_*d*_ is the desired output, and $\hat{y}$ is the estimated output of the system using the neural network.

### 5.4. Dataset

The* dataset* for the purpose of training and testing neural networks is extracted from IEEE-30 bus test case, which represents American Electric Power System. This IEEE-30 bus system contains 24 load buses, 5 PV buses, and 1 slack bus and carries details information about the buses such as generated and load powers, voltages, line admittances, and system constraints.

The inputs are real and reactive powers of the buses, which are perturbed to generate training samples, while the outputs are voltage magnitudes, phase angles, and reactive powers of the STATCOM device; the unknowns of all of them are calculated using Newton-Raphson method. The relationship between the input and output variables is highly nonlinear and therefore it requires efficient estimation techniques.

## 6. Methodology

In this study, we have estimated the output voltages, phase angles, and reactive power of the STATCOM bus using ANN with different algorithms for the training. The datasets were taken from the standard IEEE bus systems. The first step is to solve (1)–(9) using any standard numerical analysis technique such as Newton-Raphson. The Newton-Raphson is an iterative method that solves the equations iteratively using assumptions in the start. The STATCOM is connected to an appropriate load bus “*k*” and the voltage magnitude |*V*_*k*_|, phase angle *δ*_*k*_, and reactive power *Q*_*k*_ of the modified bus are also calculated using the above-mentioned equations. In order to generate large data for the STATCOM bus, the real and reactive powers of all the load buses were perturbed by increasing their values in proportions of their original values. The voltages, phase angles, and reactive powers of all the buses are calculated after each perturbation. In this way, a large dataset is obtained in which the inputs are the real and reactive powers of the buses after each perturbation, concatenated in a single column, and the outputs are corresponding values of STATCOM bus voltage magnitude |*V*_*k*_|, phase angle *δ*_*k*_, and reactive power *Q*_*k*_.

The whole dataset is randomly divided into two parts such that 70% of the data is used for the training of the ANN and the remaining 30% is used for testing. Three separate Artificial Neural Networks were used: the first one is used to estimate the voltage magnitude and the second one to estimate the phase angle of the STATCOM bus, while the third one is used to estimate the reactive power. In the standard operation, the ANN has been trained using the Back-Propagation method which is explained in the previous section. Furthermore, the ANN is also trained using Particle Swarm Optimization, Shuffled Frog Leap Algorithm, and Genetic Algorithm.

The steps for the design of neural network for each of the three cases, using metaheuristic optimization techniques, can be summarized as follows.

(1) For each case, arrange the dataset into two portions: training and testing. The total number of input features present in the dataset plays important role in determining total number of neuron in hidden layer.
(2) Initialize the total number of hidden layers and hidden neurons “*n.*” The total number of weighted neurons in the input layer is the product of number of hidden neurons “*n*” and the features of the input dataset “*m*.” Similarly, the number of total bias parameters in the hidden layer is equal to total hidden neurons, while the number of bias parameters in output layers equals the number of outputs.
(3) Calculate the total number of parameters to be optimized in the neural network. For a network with “*n*” hidden neurons and an input with “*m*” features, the total number of parameters in the input layer is “*n∗m*.” In the hidden layer, there are “*n*” weights of the neurons and “*n*” bias values. In the output layer, there are total “*o*” output bias values, corresponding to each output. Hence, the total number of parameters “*D*” to be optimized is given as
  (23) $$\begin{array}{c} D=m*n+n+n+o. \end{array}$$
(4) Initialize the PSO, SFLA, and GA populations randomly. Each solution has the dimension given by (23) and represents different combination of the weights and biases, which are explained previously. Furthermore, all the relevant constants, iterations, and constraints are initialized. Constraints must be defined according to the given conditions and can play vital role in convergence of the algorithms. Furthermore, the number of memeplexes is also defined in SFLA.
(5) The cost function is defined by (22), which is used by all the algorithms to calculate the difference between the actual output (calculated using Newton-Raphson method) and estimated output using the neural networks.
(6) For PSO, the global best *G*_best_ and *P*_best_ are calculated; the velocities and positions of particles are updated according to (16) and (17) in each iteration. For SFLA, the frogs (solutions) are ranked according to their cost and assigned to memeplexes. At each iteration, (18) to (20) are used to update the solutions. For GA, the steps for this optimization are as follows:
  (i) The parents are chosen probabilistically among the set of all solutions based on their cost with a probability.
  (ii) A crossover is applied between the two selected parents in each iteration to create a child solution. This involves selection of parents as well as the selection of split (crossover) site.
  (iii) Mutation operation takes the child and randomly changes random number of entries. This child is now placed in the population set. In the next iteration, this child would be candidate for the parent.

## 7. Results and Discussion

In order to study the efficacy of the designed STATCOM and the ANN based estimation techniques, we have tested the system on IEEE-30 bus system. The duty of STATCOM is to maintain the bus voltage at 1.0 p.u. while the estimation schemes have been used to estimate the STATCOM voltage, phase angle, and the reactive power at STATCOM. The STATCOM has been connected at the load bus 12. ANN has been trained by a variety of different optimization techniques such as Back-Propagation, PSO, SFLA, and GA. As mentioned earlier, the real and reactive powers of the IEEE-30 bus systems were perturbed by increasing the powers at the load buses by introducing a 5% change in each iteration. The corresponding voltages, phase angles, and reactive powers of the STATCOM are estimated using the Newton-Raphson method. This combination of real and reactive powers and corresponding voltages, phase angles, and reactive powers becomes the dataset for the three cases of ANN. For the purpose of summarizing the results, total 30 samples of input and output results are generated, for each of the three cases of ANNs. Out of the total dataset, a random 70% of the data is used for training, which makes a total of 21 samples, and the remaining is used for the testing purposes, which amounts to 9 samples. In all cases, the ANNs have been designed with one input layer, one hidden layer containing varying number of neurons, and one output layer containing one output. The varying number of nodes is used to consider the effect of hidden neurons in estimation. The neural network configuration is “feed forward network” with no back links. In the case of Back-Propagation training, Levenberg-Marquardt Back-Propagation algorithm is used which is the most widely used Back-Propagation method. The input contains real and reactive powers of all the buses; hence the size of input is 60. Both the real and reactive powers are concatenated in a column vector. The weights to be trained include hidden layer weights and biases plus the output layer weights and biases. In all cases of PSO, 30 initial solutions were assumed. Similarly, in the case of SFLA, a total of 50 initial solutions (frogs) have been assumed, with 10 memeplexes and 3 shuffles. In case of GA, 30 initial solutions were produced in the population. The total iterations for PSO, SFLA, and GA are fixed at 500. A summary of all the simulation parameters is depicted in Table 1.

### 7.1. Voltage Magnitude Estimation

**Figure 5:**
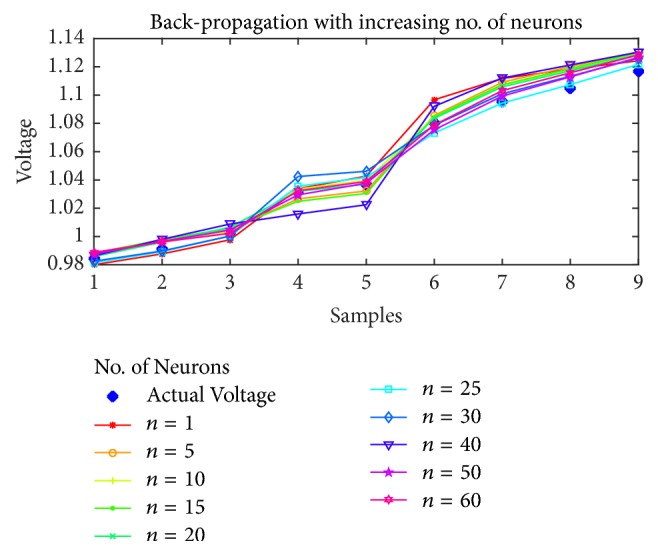
Voltage estimation using Back-Propagation with increasing number of neurons.

**Figure 6:**
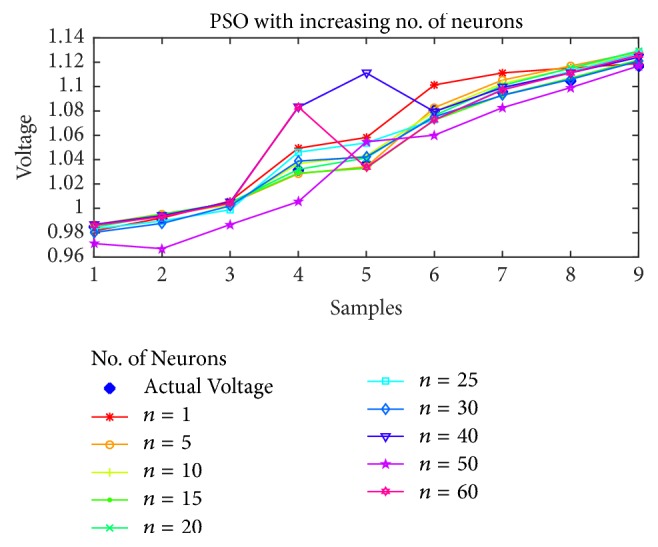
Voltage estimation using PSO with increasing number of neurons.

**Figure 7:**
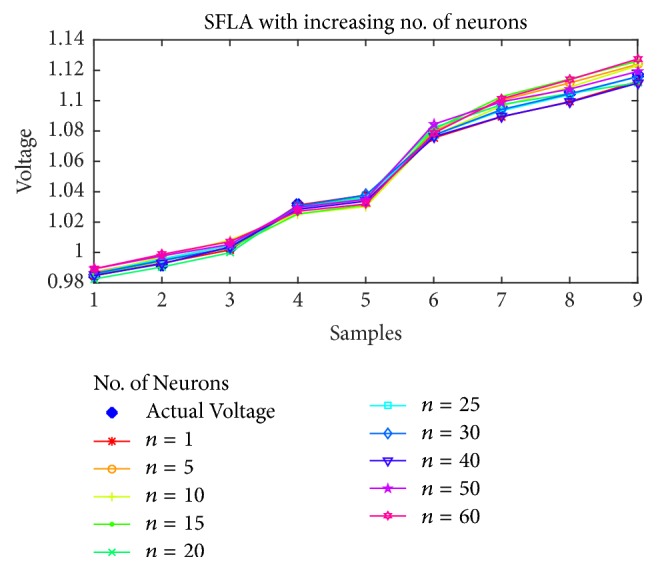
Voltage estimation using SFLA with increasing number of neurons.

**Figure 8:**
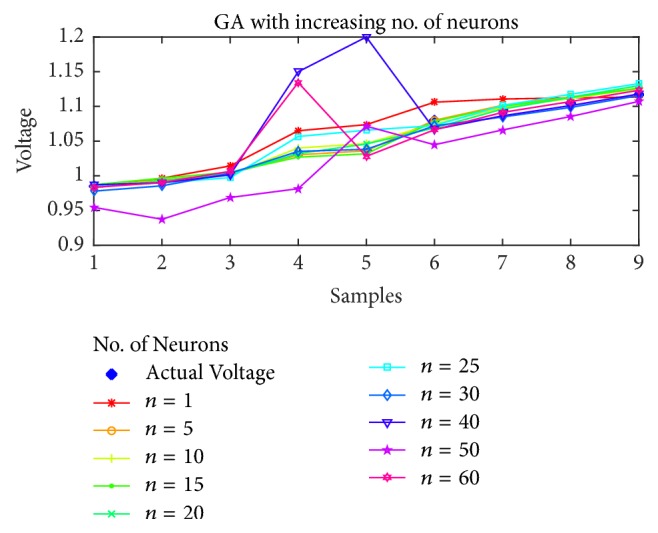
Voltage estimation using GA with increasing number of neurons.

As the first case, the voltage estimation using BP, PSO, SFLA, and GA is shown in Figures 5, 6, 7, and 8, respectively, as the number of neurons is increased from 1 to 60. It is obvious from the figures that the performance of SFLA is better than the rest in most of the cases. After SFLA, strong competition can be observed between PSO and BP algorithms. Specifically, PSO performs better than BP for intermediate number of hidden neurons in the range of *n* = 5 to *n* = 30 neurons, while BP outperforms PSO for lower and higher neurons. The reason for this behavior can be explained in terms of the fact that PSO is metaheuristic and could not find global optima when the numbers of supporting neurons was either lower or higher, while BP manages to produce slightly better results in such cases because of mathematical approach. Nevertheless, the SFLA has been able to produce lower error and better estimation, owing to its exchange of information between different memeplexes which would allow it to escape local optima more efficiently.

**Figure 9:**
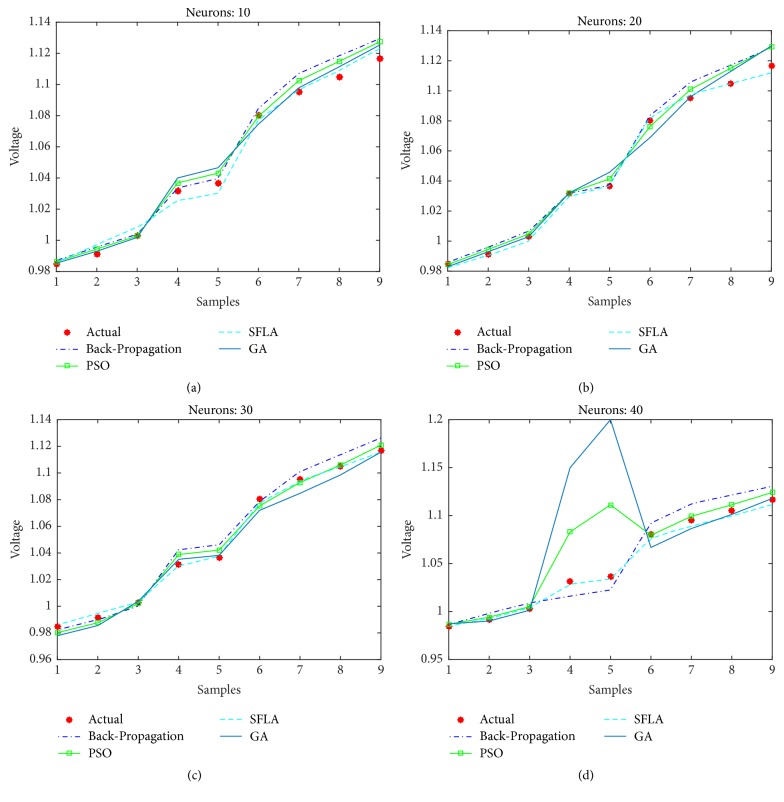
Estimation of voltages with different optimization techniques.

Furthermore, the voltage estimation of all the algorithms is also displayed for (a)* 10*, (b)* 20*, (c)* 30*, and (d)* 40* neurons in Figure 9, while the mean-squared errors for each algorithm and case are presented in Table 2.

Obviously, the SFLA has achieved mean-squared error as low as 4.99*e* − 6 for 25 hidden neurons, which implies that, with the network designed using SFLA weights, *n* = 25 would be the optimal choice for the hidden neurons. The GA performs slightly better than PSO only for lower number of neurons in the range 5–15 but produces relatively larger error for higher values of neurons. One reason for not including the results beyond *n* = 60 is the obvious stagnation or degradation in the error performance improvement as more neurons are added to the network. Therefore, it can be concluded that best choice of hidden neurons for the given dataset is between 1 and 60 neurons. The voltage estimation can directly be compared with the PSO-ANN1 voltage estimation in [53], which has used PSO to train the ANN. The estimation has rendered 2.468% error, which amounts to root mean-squared error (rmse) of 0.0719. This is higher than the maximum error in SFLA voltage estimation, whose rmse amounts to 0.0063561. For our designed network, the best estimation using SFLA is obtained with 30 neurons, as compared to 37 neurons in the PSO-ANN1. Therefore, we can conclude that SFLA based ANN with one hidden layer and 30 neurons can be used to estimate the voltage magnitude of the STATCOM bus.

### 7.2. Phase Estimation

**Figure 10:**
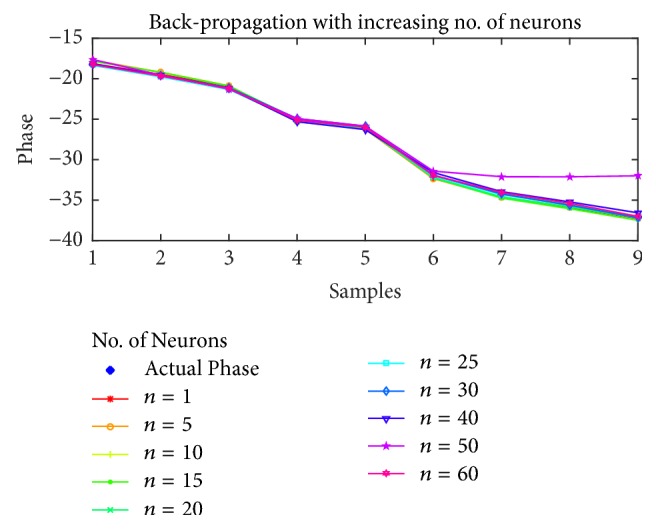
Phase estimation using BP with increasing number of neurons.

**Figure 11:**
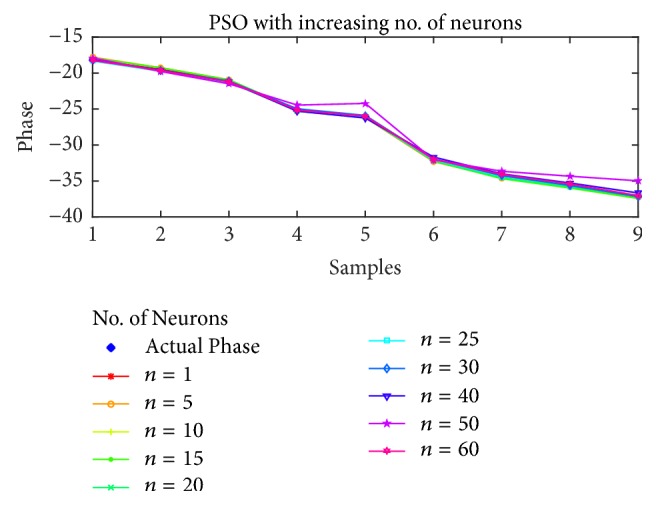
Phase estimation using PSO with increasing number of neurons.

**Figure 12:**
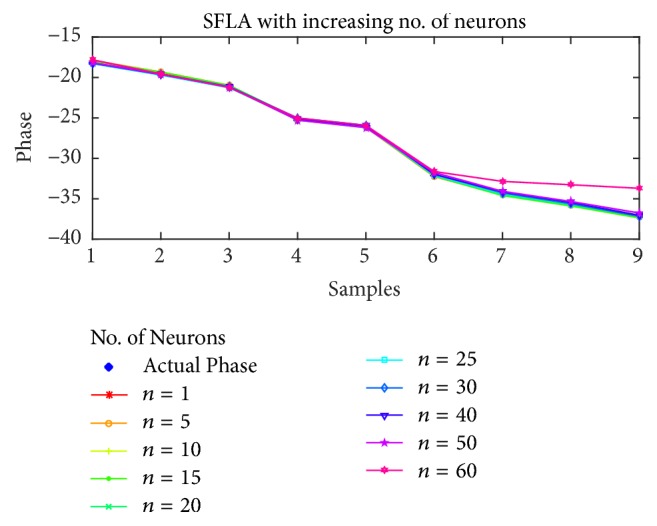
Phase estimation using SFLA with increasing number of neurons.

**Figure 13:**
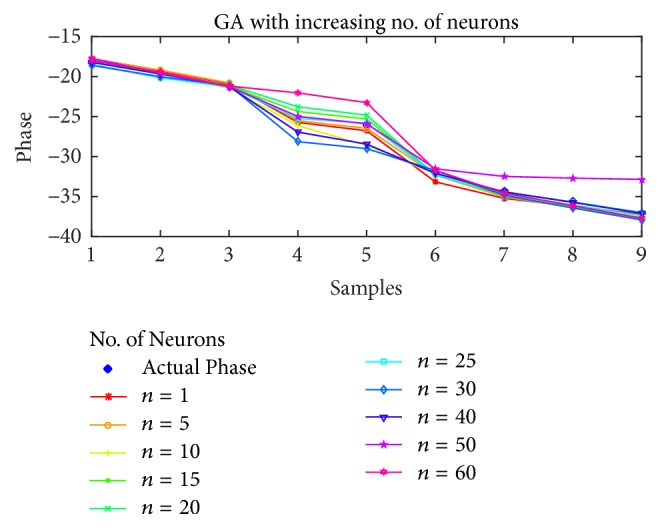
Phase estimation using GA with increasing number of neurons.

For the estimation of STATCOM phase, the results are shown in Figures 10, 11, 12, and 13 for BP, PSO, SFLA, and GA, respectively. The results show similar trend for all the optimization techniques as in the case of voltage estimation. Usually, the error is minimum in range of 10 to 40 neurons for all the optimization techniques. For BP and PSO, the best estimation is observed at *n* = 10, although more consistent results can be observed in the range of 20 to 30 neurons. For SFLA, similar trend is observed with optimal results at *n* = 10, which is also the overall minimum MSE. It is also evident that beyond hidden neurons greater than 40, the error can be seen to rise and produce poor estimation. We have included results up to 60 neurons due to limited space.

**Figure 14:**
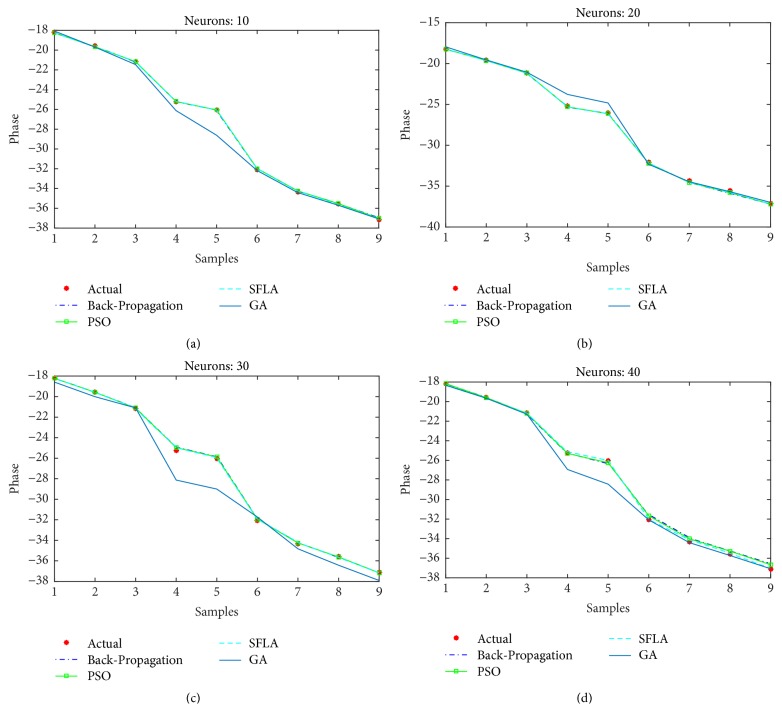
Estimation of phases with different optimization techniques.

Furthermore, the phase estimation of all the algorithms is also displayed for (a)* 10*, (b)* 20*, (c)* 30*, and (d)* 40* neurons in Figure 14, while the mean-squared errors for each algorithm and case are presented in Table 3. The MSE are comparatively higher than the previous case of voltage estimation. This might partly be due to the regression which involves wider range of floating number as compared to the previous case, while the nonlinearity of the estimation might also have played its role. Nevertheless, the SFLA still outperforms other algorithms in terms of MSE. The distribution of frogs into memeplexes and each frog constructively improving the ideas of its memeplex independent of other memeplexes can lead to better results when compared with other algorithms. On the contrary, the GA is not proved efficient in the estimation of phase for which several reasons can be presented such as overfitting on the training data, unguided mutation, and its appropriateness for binary based problems.

### 7.3. Reactive Power Estimation

**Figure 15:**
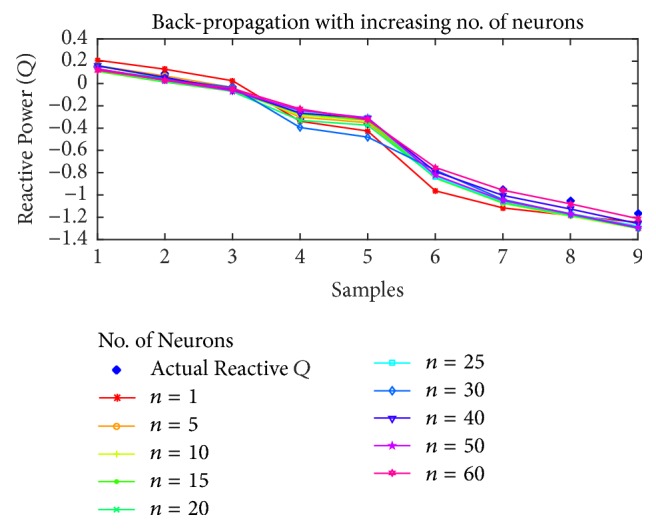
Reactive power (*Q*) estimation using Back-Propagation with increasing number of neurons.

**Figure 16:**
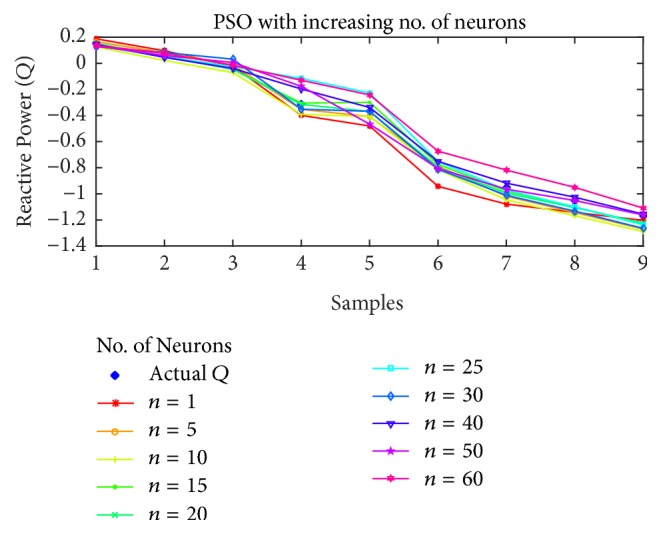
Reactive power (*Q*) estimation using PSO with increasing number of neurons.

**Figure 17:**
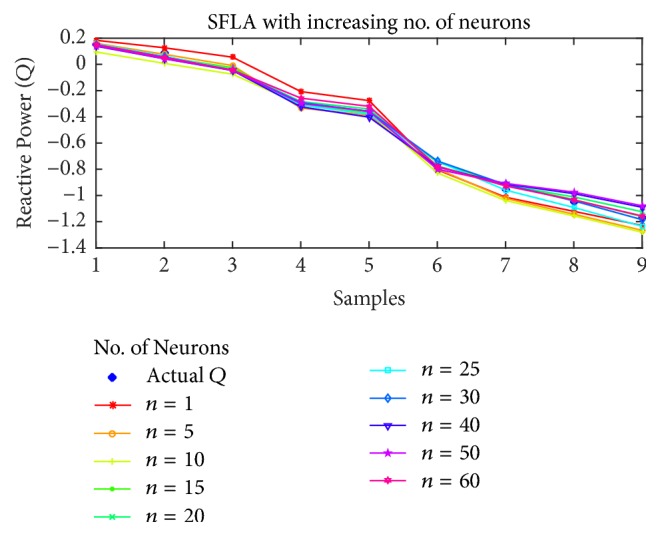
Reactive power (*Q*) estimation using SFLA with increasing number of neurons.

**Figure 18:**
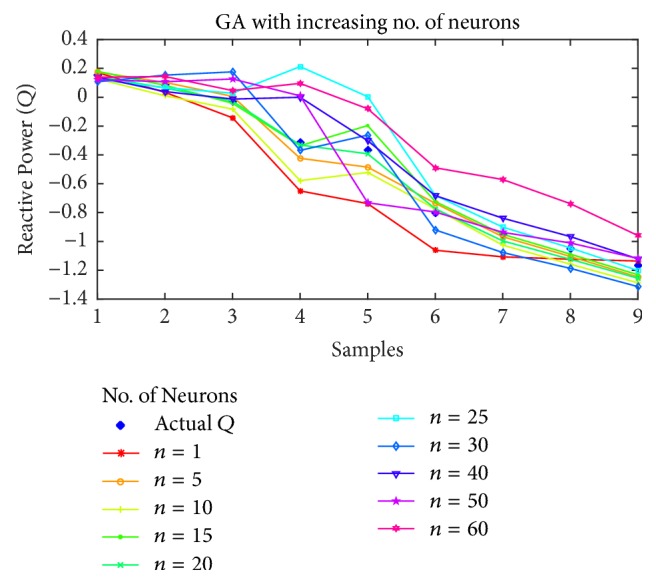
Reactive power (*Q*) estimation using GA with increasing number of neurons.

For reactive power estimation, the results are shown in Figures 15, 16, 17, and 18 for BP, PSO, SFLA and GA, respectively. As opposed to the previous estimations, the lower number of neurons has resulted in higher than usual error, while the minimum error shift is not homogenous, although it can be noted that the error is minimum in the mediocre range of neurons. For BP, the best estimation is achieved at *n* = 40 and then error rises again. For PSO, the minimum error seems to oscillate with the increase in neurons, but lowest error is attained at *n* = 20, followed by 15 and 40. For SFLA, the minimum error period is more consistent in the range of 15 to 30, in which minimum error is observed for the estimation of reactive power. In fact, the SFLA still produces the best estimation of the reactive power, while comparison also depicts the better performance of PSO when compared to the BP, which is again attributed to the superiority of metaheuristic approaches in finding the global optima. As opposed to the previous case, it can also be seen that GA seems to perform on par with the BP in estimation when the number of neurons is between 5 and 20. But for higher number of neurons, GA fails to perform best.

**Figure 19:**
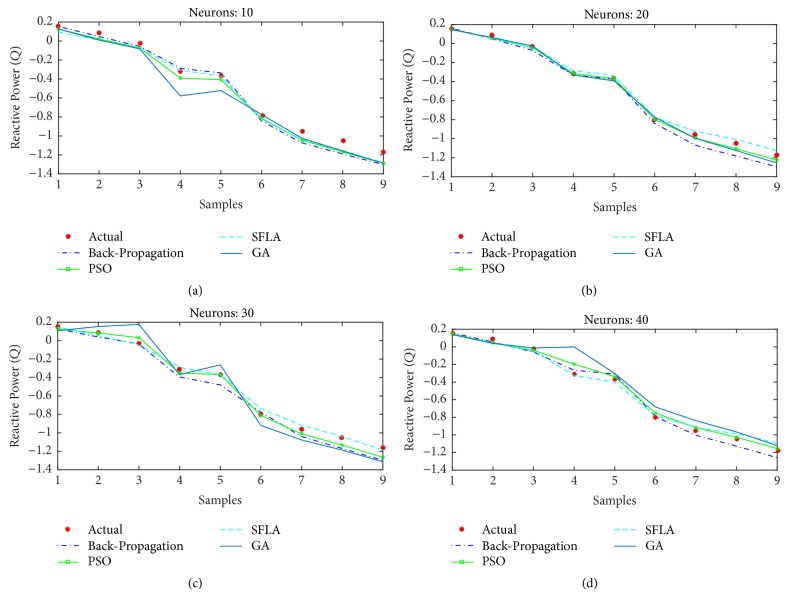
Estimation of reactive power (*Q*) with different optimization techniques.

As in the previous cases, the reactive power estimation of all the algorithms is also displayed for (a)* 10*, (b)* 20*, (c)* 30*, and (d)* 40* neurons in Figure 19, while the mean-squared errors for each algorithm and case are presented in Table 4. Without any effort, it can be concluded that SFLA's estimation is best in reactive power estimation, closely followed by the PSO. The constant failing of GA sheds light on the fact that it does not perform well in regression problems and it is more suitable for scheduled optimization.

This estimation can be applied to larger power systems such as IEEE-300 bus system to understand the dynamics of larger amount of data and its effect on different optimization techniques.

## 8. Conclusion

This paper is focused on fine-tuning the weights of Artificial Neural Network (ANN) using different optimization techniques. Then, the developed ANN is used to estimate voltages, phases, and reactive powers of STATCOM. We have discussed the role of STATCOM in power buses for the voltage regulation of the buses. We have presented an efficient iterative method to solve the power systems which involve STATCOM. The STATCOM introduces some new variables in the system which must be accounted for. In the next stage, we have provided an alternative method to calculate the unknown variables in STATCOM using Artificial Neural Network curve fitting, which is faster and requires less memory and processing power. We have trained Artificial Neural Networks with different optimization techniques such as Back-Propagation, Particle Swarm Optimization, Shuffled Frog Leap Algorithm, and Genetic Algorithm. The results show that Shuffled Frog Leap Algorithm can perform better than the Back-Propagation and other methods when the parameters are fine-tuned according to the given dataset, while PSO and BP performance are also acceptable.

## Figures and Tables

**Table 1:** Summary of simulation parameters.

Network	Feed-forward
Number of networks	3
Optimization techniques	Back-Propagation, PSO, SFLA, GA
Neurons	1, 5, 10, 15, 20, 25, 30, 40, 50, 60
Hidden layers	1
Input size	60
Output size	1
Inputs	Real and reactive powers of load buses
Outputs	STATCOM voltage, phase and reactive power

**Table 2:** MSE in voltage estimation.

*n*	Back-Propagation	PSO	SFLA	GA
1	9.79*E* − 05	0.00018	1.25*E − *05	0.000403
5	8.04*E* − 05	4.66*E* − 05	1.75*E* − 05	2.36*E* − 05
10	6*E* − 05	3.86*E* − 05	2.31*E* − 05	3.7*E* − 05
15	7.45*E* − 05	1.51*E* − 05	3.38*E* − 05	2.84*E* − 05
20	4.99*E* − 05	3.84*E* − 05	5.61*E* − 06	5.11*E* − 05
25	1.62*E* − 05	8.47*E* − 05	4.99*E* − 06	0.000226
30	4.66*E* − 05	1.88*E* − 05	3.01*E* − 06	3.56*E* − 05
40	0.000155	0.000928	1.44*E* − 05	0.004558
50	2.71*E* − 05	0.000294	1.34*E* − 05	0.001255
60	4.03*E* − 05	0.000322	4.04*E* − 05	0.001214

**Table 3:** MSE in phase estimation.

*n*	Back-Propagation	PSO	SFLA	GA
1	0.038329	0.026473	0.016814	0.389082
5	0.081626	0.056788	0.036451	0.239865
10	0.008381	0.005758	0.003634	0.83136
15	0.104433	0.072338	0.046123	0.284553
20	0.019951	0.013768	0.008738	0.424073
25	0.013029	0.009192	0.006031	0.104451
30	0.019706	0.014014	0.009299	2.128638
40	0.100567	0.069504	0.009352	0.958293
50	4.929065	1.213655	0.044166	3.422461
60	0.019833	0.014103	2.189899	2.099536

**Table 4:** MSE in reactive power estimation.

*n*	Back-Propagation	PSO	SFLA	GA
1	0.009706	0.007413	0.004553	0.040481
5	0.005537	0.003064	0.002702	0.004669
10	0.00643	0.005783	0.004836	0.015066
15	0.006869	0.001567	0.000667	0.004621
20	0.00576	0.001045	0.000894	0.001944
25	0.00562	0.007867	0.001253	0.048621
30	0.006889	0.002816	0.000889	0.014558
40	0.002592	0.002344	0.00182	0.015827
50	0.00579	0.003539	0.002045	0.029956
60	0.004101	0.010924	0.000926	0.071632

## Supplementary Materials

Supplementary Materials Supplementary materials contain a short video-clip about the code, which was adopted to simulate the performance of Neural Networks with different optimization techniques.
